# Neutrophil Extracellular Trap Density Increases With Increasing Histopathological Severity of Crohn’s Disease

**DOI:** 10.1093/ibd/izab239

**Published:** 2021-11-01

**Authors:** Angie L Schroder, Belal Chami, Yuyang Liu, Chloe M Doyle, Mary El Kazzi, Golo Ahlenstiel, Gulfam Ahmad, Nimalan Pathma-Nathan, Geoff Collins, James Toh, Andrew Harman, Scott Byrne, Grahame Ctercteko, Paul K Witting

**Affiliations:** 1 The University of Sydney, School of Medical Sciences, Faculty of Medicine and Health, NSW, Australia; 2 Charles Perkins Centre, The University of Sydney, NSW, Australia; 3 Westmead Institute for Medical Research, Centre for Immunology and Allergy Research, Westmead, NSW, Australia; 4 Western Sydney University, Westmead Clinical School and The Westmead Institute for Medical Research, Blacktown Hospital, Blacktown, NSW, Australia; 5 Centre for Virus Research, The Westmead Institute for Medical Research, Westmead, NSW,Australia; 6 Department of Colorectal Surgery, Westmead Hospital, NSW,Australia

**Keywords:** inflammatory bowel disease, myeloperoxidase, neutrophil extracellular traps

## Abstract

**Background:**

Intestinal neutrophil recruitment is a characteristic feature of the earliest stages of inflammatory bowel disease (IBD). Neutrophil elastase (NE) and myeloperoxidase (MPO) mediate the formation of neutrophil extracellular traps (NETs); NETs produce the bactericidal oxidant hypochlorous acid (HOCl), causing host tissue damage when unregulated. The project *aim* was to investigate the relationship between NET formation and clinical IBD in humans.

**Methods:**

Human intestinal biopsies were collected from Crohn’s disease (CD) patients, endoscopically categorized as unaffected, transitional, or diseased, and assigned a histopathological score.

**Results:**

A significant linear correlation was identified between pathological score and cell viability (TUNEL^+^). Immunohistochemical analysis revealed the presence of NET markers NE, MPO, and citrullinated histone (CitH3) that increased significantly with increasing histopathological score. Diseased specimens showed greater MPO^+^-immunostaining than control (*P* < .0001) and unaffected CD (*P* < .0001), with transitional CD specimens also showing greater staining than controls (*P* < .05) and unaffected CD (*P* < .05). Similarly, NE^+^-immunostaining was elevated significantly in diseased CD than controls (*P* < .0001) and unaffected CD (*P* < .0001) and was significantly higher in transitional CD than in controls (*P* < .0001) and unaffected CD (*P* < .0001). The CitH3^+^-immunostaining of diseased CD was significantly higher than controls (*P* < .05), unaffected CD (*P* < .0001) and transitional CD (*P* < .05), with transitional CD specimens showing greater staining than unaffected CD (*P* < .01). Multiplex immunohistochemistry with z-stacking revealed colocalization of NE, MPO, CitH3, and DAPI (cell nuclei), confirming the NET assignment.

**Conclusion:**

These data indicate an association between increased NET formation and CD severity, potentially due to excessive MPO-mediated HOCl production in the extracellular domain, causing host tissue damage that exacerbates CD.

## Graphical Abstract

**Graphical Abstract:**
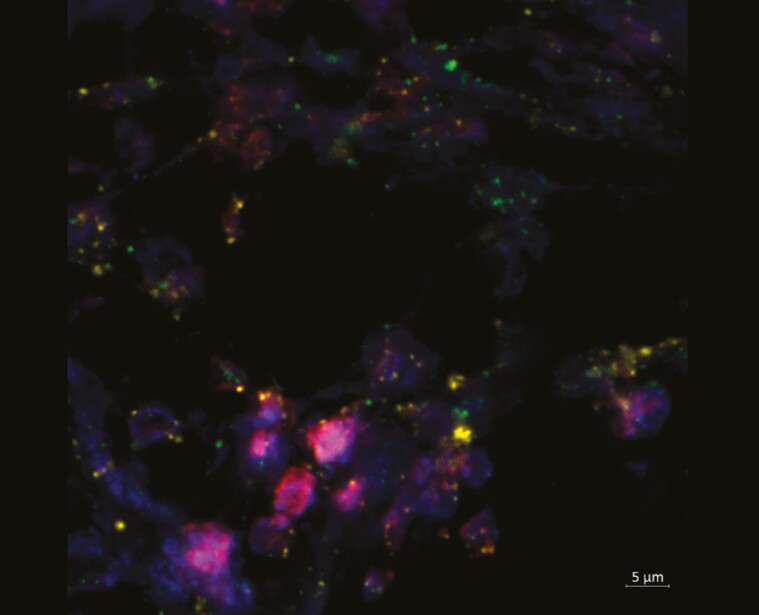

## Introduction

Crohn’s disease (CD) is characterized by intermittent chronic inflammatory lesions throughout the gastrointestinal (GI) tract.^1,2^ It is a subtype of the broader category of Inflammatory Bowel Disease (IBD), which also includes ulcerative colitis (UC).^1^ Unlike UC, transmural lesions are common in CD, with loss of crypt density and height and erosion of villi characteristic of the damage to colon architecture. In addition, granuloma formation is common, with evidence of excessive infiltration by inflammatory cells in the histologically confirmed disease-active regions together with basal plasmacytosis and invasion of gut flora into the colon wall.^3^ Macroscopic features include a “cobblestone” appearance of the mucosa, bowel adhesions, and fistula formation in extreme cases of inflammation.^4^

Neutrophils are among the first cell types recruited to tissue during pathogen insult; neutrophils play a role in first-line defence by regulating the local host inflammatory response as part of a crucial balanced innate response to pathogen management. The subsequent activation of neutrophils following recruitment results in the release of chemical mediators that are in turn involved in the recruitment of other immune cells that assist in the resolution phase of inflammation.^5^ Although their response is beneficial, excessive neutrophil recruitment and accumulation can lead to mucosal injury and increased bowel permeability to bacteria,^6^ which is characteristic feature of CD.

The chronic inflammatory nature of IBD is thought to be perpetuated due to dysfunctional immune and barrier mechanisms in the bowel, resulting in irreversible structural and functional changes from the prolonged inflammatory response.^7^ Cellular and tissue damage is mediated through the production of reactive oxygen species (ROS) primarily generated from recruited innate immune cells, including hydrogen peroxide (H_2_O_2_), superoxide radical anion (O_2_^•-^), hydroxyl radical (HO^•^), and hypochlorous acid (HOCl).^8^ The production of ROS at sites of inflammation elicits an antimicrobial activity via the nonspecific oxidative damage of DNA, lipids, and proteins in the invading pathogen. However, unregulated ROS production is also linked to oxidative host-tissue damage^9^ that promotes further inflammatory cell recruitment, resulting in a cyclic loop of chronic inflammation.^10^ A balance of ROS production and endogenous antioxidants is crucial for normal inflammatory activity and pathogen clearance in the gut barrier^11^; an imbalance can result in chronic tissue damage.

The potent 2-electron oxidant HOCl is produced via the halogenation cycle of the neutrophil enzyme myeloperoxidase (MPO).^12^ Intracellular MPO is stored in azurophilic granules, whereby following phagocytosis, MPO-containing azurophilic granules fuse with the phagosome to form a phagolysosome to initiate intracellular destruction of ingested microbes.^13^ Recent evidence indicates that MPO can also be released from activated neutrophils to eradicate bacteria in the extracellular space^14^ and *neutrophil extracellular traps* (NETs) became a term coined to explain neutrophil extracellular bactericidal activity; it was later revealed MPO was fundamental to NET formation.^15,16^ For example, NET release is dependent on the oxidative burst and HOCl production via MPO.^17^ Inhibiting activity of nicotinamide adenine dinucleotide phosphate (NADPH) oxidase, an enzyme required for the oxidative burst, was shown to impair NET formation in stimulated neutrophils, with addition of glucose oxidase to bypass the requirement of NADPH oxidase, resulting in restoration of NET formation.^18^ Inhibition of MPO activity also results in poor NET formation, with neutrophils isolated from MPO-deficient patients shown to be unable to form NETs, while addition of exogenous MPO was unable to restore NET formation.^15^ Notably, correlations between MPO and IBD disease severity was first identified in 1998, with grading of lesions viewed endoscopically shown to correlate to patient stool concentration of MPO.^19^ Furthermore, endoscopic analysis of UC lesion showed improved colon health after treatment with prednisolone, which correlated with a decrease in bowel intraluminal MPO concentration.^20^

Although NETosis was first described over a decade ago, it was only recently identified in patients with IBD.^21^ Thus, NET formation begins with activation of peptidylarginine deiminase type IV (PAD4), a granular and nuclear enzyme which converts arginine residues to citrulline when activated.^22^ Chromatin becomes decondensed when histones bound to DNA in the nucleus are citrullinated, particularly histone H3 (CitH3).^23^ Subsequent disintegration of the nuclear membrane allows intracellular granular enzymes to mix with the decondensed genetic material,^24,25^ including neutrophil elastase (NE), a serine protease with broad specificity that enables nonspecific bacterial clearance via destruction of virulence factors on the cell membrane.^26^ When the neutrophil cell membrane ultimately bursts, the conglomeration of cellular material enters the extracellular space, with the resulting filamentous structure porous enough to trap a variety of pathogens while allowing small molecules to pass through.^17^ Overall, trapping and killing of bacteria continues after neutrophil death.

Formation of NETs has been observed in pediatric IBD cases,^27^ with both CD and UC cases displaying NETosis defined through immunohistochemical analysis of NET markers. In support of NETosis in adult IBD, metaproteomic analysis of fecal samples has demonstrated increased concentration of NET-associated proteins in both UC and CD patients.^28^ Notably, identifying NET formation in adult tissue remains to be unambiguously defined; the available data were obtained using a histone marker that is not specific to NETosis.^27^

Although recent reports have demonstrated the presence of NETS in human IBD colonic samples, no studies have quantified individual components of NETs in relation to the histopathologically affected ileal biopsies from clinically confirmed CD patients. We demonstrate the presence of ileal NET structures using Opal multi-plex-enabled 4-plex immunohistochemistry for MPO, neutrophil elastase, citrullinated histone 3, and DNA-visualized through a high-resolution confocal LSM−800 microscopy, with Airyscan module to obtain 3D structure of NETS.

## Materials and Methods

### Materials

All standard laboratory reagents and biochemicals were purchased from Sigma Aldrich, Australia (unless otherwise stated) and were of the highest quality available. All reactions and washing steps were performed at 22ºC and in tris buffered saline containing 5% (w/v) tween-20 (TBS-T), respectively, unless otherwise specified.

### Methods

#### Patient cohort

Patients previously diagnosed with CD who were scheduled to undergo routine endoscopy or surgery were identified as suitable candidates to provide biopsy samples for research purposes. Written consent was obtained as per the approved human ethics protocol. During the procedure, 3 individual biopsy samples were collected from the ileum of each patient (Table 1) from regions determined visually to be

**Table 1.** Description of the ileal sampling and initial endoscopic characterization of biopsy tissue.

Prognosis^a^	De-identified	Samples	Region/	Age	Sex	Pathological featuresb
	Patient ID	Provided	Condition			
Healthy Control	190308	1	Healthy	66	M	Ileostomy reversal—some fibrosis at edges of specimen from prior surgery, mucosa elsewhere is unremarkable.
	190527	1	Healthy	74	M	N/A^c^
	241018	1	Healthy	56	F	Right hemicolectomy—removal of fungating tumour extending into muscularis propria. No evidence of metastasis in lymph nodes. Pathological changes isolated to tumour.
Crohn’s Disease	030818	2	Unaffected, Transitional	78	M	Right hemicolectomy—terminal ileum shows cobblestoning and polyps.
	031018	3	Unaffected, Transitional, Diseased	18	M	Right hemicolectomy—stenosis with mesenteric fat creeping. Thickened bowel wall with cobblestoning and congestion. Muscularisation of the submucosa and fibrosis of pericolic fat.
	100519	2	Unaffected, Diseased	60	M	Ileocolic resection—extensive thickening of intestinal wall, and serosal fat creeping. Ulceration of mucosa.
	100818	3	Unaffected, Transitional, Diseased	29	M	Ileocecal resection—wall thickening, with exudate and haemorrhagic appearance of serosa. Adhesion of adjacent sections of small bowel due to deep fissuring abscess. Granulomas and submucosal fibrosis present.
	180918	3	Unaffected, Transitional, Diseased	25	M	N/A^d^
	281118	3	Unaffected, Transitional, Diseased	50	F	Right hemicolectomy—serosal fat wrapping and small adhesions. Thickened bowel wall with stricture formation proximal to ileocecal valve. Ulceration and cobblestoning of mucosa, with possible pseudopolyp formation. Villous blunting and fissuring of mucosa.

^a^Prognosis for healthy controls and Crohn’s disease patients was determined through a combination of microscopic and macroscopic features observed upon endoscopy, biopsy collection, imaging and clinical history.

^b^Identified at time of operation. Final histological analysis/scoring was performed “in-house” at a later date.

^c^Patient underwent stoma surgery with no pathology report generated.

^d^Pathology report not available due to surgical procedure performed at a private hospital.

1) free of active lesions and essentially free of inflammatory disease (designated as unaffected),
2) adjacent to disease lesion tissue in the region, before obvious disease was visually identified (designated as transitional), and
3) an active disease lesion tissue with obvious signs of inflammation (designated as diseased).

After collection, the tissue was placed in a plastic cassette containing Optimal Cutting Temperature (OCT; Tissue Tek, USA) embedding medium, then snap frozen in liquid nitrogen, and transferred to long-term storage at −80ºC in the tissue Biobank (Discipline of Surgery, Westmead Hospital). An ex vivo tissue biopsy was also performed on previously excised ileal tissue taken from patients either undergoing intestinal resectioning or ileostomy surgery. In this case, tissue was taken from the extreme margin of the excised portion and assigned as healthy intestine by the attending surgeon. These specimens were designated as control tissue for comparative studies and stored in identical fashion to the other isolated samples. Where required, fresh-frozen specimens were transported on dry ice from the Biobank to the Redox Biology Lab, Charles Perkins Centre, The University of Sydney.

### Ethics Statement

Small bowel excisions were acquired from patients admitted to Westmead Adult Hospital, Sydney, Australia, by an experienced general surgeon under current Human Research Ethics Codes (HRECs; Westmead Biobank approval HREC#4192; HREC/#15; WMEAD/#11). All biopsy samples of human intestine were taken from patients undergoing endoscopy after obtaining informed consent prior to long-term storage at −80ºC in the Westmead Biobank (Discipline of Surgery, Westmead Hospital).

#### Tissue sectioning

Excised bowel samples were embedded in OCT and serially sectioned at 7-µm thickness using a cryostat for which the chamber was set at −20˚C and sample set at −12˚C. Sectioned tissue was collected onto Superfrost Plus glass slides (Menzel-Gläser, Germany) and air-dried for 30 minutes. Slides were then stored at −80˚C until required.

#### Hematoxylin and eosin staining

Slides were retrieved from −80˚C storage and allowed to thaw at 22˚C for 10 minutes before fixation with prechilled 100% methanol for 5 minutes. Slides were then washed for 1 minute under running tap water and immersed in Harris’s hematoxylin for 2 minutes before being washed and differentiated in 0.25% (v/v) acid alcohol/water solution. Slides were briefly rinsed in tap water then immersed in Scott’s blue solution for 30 seconds, rinsed, and dehydrated in 70% (v/v) alcohol. Treated sections were then counterstained in eosin (2 × 40 seconds immersions) and subsequently dehydrated through graded alcohol solutions before being cleared in xylene and cover-slipped with resinous dibutylphthalate polystyrene xylene (DPX, mounting medium; Sigma-Aldrich, 06522).

#### Alcian blue and acetic safranin staining

Air-dried slides were fixed in 10% (w/v) neutral buffered formalin for 10 minutes and washed under running tap water for 1 minute. The slides were immersed in Alcian blue solution (1%, pH 2.5) for 30 minutes and subsequently washed in tap water again. Sections were then counterstained (5 minutes, 0.1% w/v acetic safranin solution), then washed and left to air dry for 30 minutes. The slides were then cover-slipped with DPX as described previously.

#### Histopathological scoring

Stained sections were viewed using Zeiss Axio Scope A.1 microscope under 10x objective magnification and photographed using Zen 2 (Blue edition) software, with the researcher performing the histological scoring blinded to the specimen. Fields of view between sections of each sample stained with hematoxylin and eosin (H&E) and Alcian blue were carefully matched and imaged at 10 × 10 magnification. For each section, 3 to 20 images were required to completely capture the entire surface area of each section. Scoring of disease severity was performed using H&E and Alcian blue stained sections by a single, blinded pathologist using a previously established grading tool^29^ that accounts for the following criteria: crypt structural loss, infiltration of leukocytes, epithelial integrity, goblet cell loss, and edema, as previously used to determine pathological scoring (detailed histological criteria described in Supplementary Table 1). Individual criteria were scored on a scale from 0 (unaffected) to 2 (completely affected) to generate a cumulative score (maximum value 10) for each field of view. The total score for each sample was then normalized against the number of fields of view to create an average pathological score for each sample. The process of scoring was performed to confirm that endoscopic classification of the tissue matched with the extent of inflammatory damage seen in the tissue. Through undertaking this histological assessment, it became apparent that it was necessary to reclassify 2 biopsy specimens initially designated as unaffected CD samples by endoscopic inspection, as there was clear evidence of active inflammation within the sampled tissue. As all unaffected samples were taken relatively nearby a lesion due to the constraints of endoscopy, the 2 samples were reclassified to the transitional category to account for this complication. This approach resulted in the following sample numbers in each confirmed category:

1) Unaffected (0–4, *n *= 5)
2) Transitional (4.5–7, *n* = 6)
3) Diseased (7.5–10, *n* = 5)
4) Healthy control (<4, *n* = 3)

#### Assessment of cell viability with TUNEL

Cell viability (taken as a surrogate marker to indicate the end point of inflammatory injury) was measured using a DeadEnd Fluorometric TdT-mediated dUTP Nick-End Labeling (TUNEL) kit (Promega, G3250). Tissue sections were thawed (22ºC) and then air-dried for 30 minutes prior to fixation (15 min) in 4% (v/v) formaldehyde (Thermo Fisher Scientific, 28908, diluted in 10 mM of phosphate buffered saline [PBS]). Following consecutive washes in PBS, slides were incubated with 20 µg/L of proteinase K solution (9.5 min, 22ºC) and then washed in PBS before fixation (5 min) in 4% (v/v) formaldehyde in PBS. Next, the tissue sections were incubated with equilibration buffer for 10 minutes, after which TdT reaction solution was added, and the mixture was incubated further (37ºC, 60 min). Next, the slides were immersed in 2x saline-sodium citrate buffer for 15 minutes, after which they underwent 3x consecutive washes in PBS. Finally, sections were counterstained with 4’,6-diamidino-2-phenylindole (DAPI; PerkinElmer, FP1490), washed and cover-slipped with fluorescence mounting medium (Agilent Technology, S3023), and stored in the dark prior to imaging.

#### Optimizing single-plex immunofluorescence imaging

Serially cut frozen slides were air-dried and fixed in prechilled 100% acetone). Slides were rinsed in distilled water and then washed in TBS-T. Endogenous peroxidase activity was quenched via incubation with 5% (v/v) H_2_O_2_ (Sigma-Aldrich, H3410) in dH_2_O. After 30 minutes, the slides were washed and incubated in serum-free protein block (DAKO, X0909) for 30 minutes, after which excess liquid was removed. Serial sections were then incubated with 1:1000 (v/v) anti-MPO (mouse, monoclonal; Abcam, ab25989) or 1:1000 (v/v) anti-NE (rabbit, monoclonal; Abcam, ab131260) or 1:500 (v/v) anti-CitH3 (rabbit, monoclonal; Abcam, ab219407) for 1 hour in antibody diluent (bovine serum albumin [1% w/v], Sigma-Aldrich, A7906) and Triton-X100 (0.5% v/v, Sigma-Aldrich, 270733) in TBS-T. Slides were further washed in TBS-T and incubated with horse radish peroxidase (HRP)-coupled secondary antibody (anti-mouse, DAKO, K4001; anti-rabbit, K4003) for 30 minutes. Following washing in TBS-T, slides were incubated for 10 minutes with Opal690 fluorophore (PerkinElmer, FP1497) in tyramide signal amplification (TSA) buffer (PerkinElmer, FP1498, 1:50 v/v dilution in 1x plus amplification buffer) and subsequently washed. Specimens were incubated in a 1:800 (v/v) dilution of Spectral DAPI for 10 minutes, washed, and then cover-slipped with fluorescence mounting medium.

#### Opal multi-plex immunofluorescence

Detection of multiple targets on tissue sections was achieved through application of the Opal kit developed by PerkinElmer. The kit allows fluorescent labeling of up to 7 targets via TSA, in which a fluorescent-labeled tyramide substrate is converted by added horse radish peroxidase into a form able to bind to protein tyrosine residues at or near the HRP-labeled antibody-antigen complex. Microwave treatment then cleaved the antibody complex, leaving the fluorescent-labeled tyramide intact and enabling further antibody treatment for other immune-active targets on the same tissue section.

Briefly, serially cut frozen slides were air-dried and fixed in 100% acetone (−30ºC). Slides were rinsed in distilled water, then submerged in 10 mM of PBS and microwaved at 1000w power until boiling, then at 20% power for 20 minutes. Endogenous peroxidase activity was quenched via incubation with 5% (v/v) H_2_O_2_ (Sigma-Aldrich, H3410) in dH_2_O for 30 minutes. Slides were washed and incubated in serum-free protein block (DAKO, X0909) for 30 minutes, after which excess liquid was removed. Primary antibodies, including anti-NE (PerkinElmer, FP1487, final dilution 1:1000 v/v), anti-MPO (PerkinElmer, FP1488, final dilution 1:1000 v/v), and anti-CitH3 (PerkinElmer, FP1497, final dilution 1:500 v/v) were incubated during separate microwave cycles for 1 hour. Following TBS-T washing, peroxidase conjugated secondary antibody (antimouse, DAKO, K4001; antirabbit, K4003) was incubated for 30 minutes. Fluorescent-labeled tyramide substrate (1:50 v/v) was then incubated for 10 minutes in the following combinations: NE, Opal520; MPO, Opal570; CitH3, Opal690. Specimens were incubated in a 1:800 (v/v) dilution of Spectral DAPI for 10 minutes, washed, and then cover-slipped with fluorescence mounting medium.

#### Imaging equipment and quantification of staining

All TUNEL and single-plex immunoreactive signals were captured at 10x magnification under the same exposure settings using an AxioCam ICm 1 monochromatic camera connected to an Axio Scope.A1 microscope. Images were captured using ZEN blue imaging software (2nd edition) and exported as .TIFF files for analysis. Each specimen had 3 to 5 fields of view captured of the mucosa, with quantification performed using FIJI (version 2.0.0-rc-69/1.52p). The freehand selection tool was used to create a region of interest around the tissue border using the DAPI-stained section image, which was then copied and overlayed across the corresponding antibody-stained section image, thereby representing the desired region of analysis for each individual tissue section. A maximum threshold was set to capture the mean grey value of each pixel, and the overall mean grey value was determined against the number of regions of interest.

#### Multiplex imaging

Opal slide imaging was performed on an LSM 880 confocal microscope with Airyscan processing (Zeiss, Australia), with images captured at 40x magnification. Background light is omitted during imaging through the multichannel area detector with 32 sensors that act as virtual pinholes. The microscope uses a main beam splitter (MBS) and an emission filter (bandpass or longpass) to isolate and detect fluorescence from each individual fluorophore. A combination of DAPI and Opal fluorophores 520, 570, and 690 were imaged under the conditions outlined in Table 2. Images were generated by selecting the boundaries of the tissue on the z-axis, followed by sequentially capturing images at several focal distances to generate a 3D image of the entire sample depth.

**Table 2.** Commercial fluorophores used in multiplex image capture experiments.

Fluorophore	Excitation Wavelength	Detection (Emission) Wavelength	Main Beam splitter
DAPI	405 nm	450 nm	MBS 405
Opal 520	488 nm	516 nm	MBS 488/561/633
Opal 570	561 nm	579 nm	MBS 488/561/633
Opal 690	633 nm	654 nm	MBS 488/561/633 + LP 650

#### Statistical analysis

Statistical analysis of data was performed using GraphPad Prism (version 8.2.0). Data are expressed as the mean ± SD. Prior to undertaking analyses, total data sets were judged to be normally distributed as assessed by the Shapiro-Wilk test and, as such, were analyzed using the Pearson correlation coefficient. However, when grouped in their respective pathological categories, the data subsets were nonparametrically distributed, thus the Kruskal-Wallis test was performed under circumstances where between-group comparisons were made. Results that corresponded to values of *P* ≤ .05 were considered statistically significant.

## Results

### Validating the Severity of Bowel Inflammation

**Figure 1.**
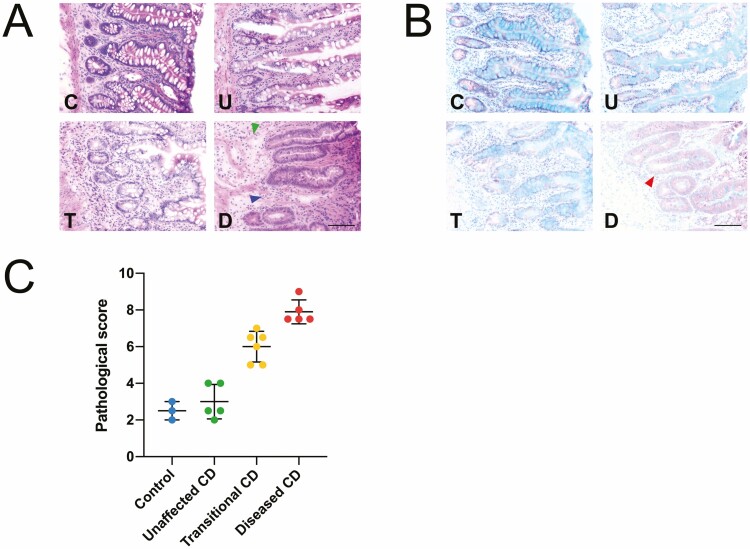
Representative histological stained biopsy samples stained with Hematoxylin & Eosin (A) and Alcian blue (B). Images are of control tissue (C), unaffected (U), transitional (T), and diseased (D) Crohn’s disease tissue. Blue arrowhead indicates crypt dropout, green indicates edematous disruption of basement membrane, red indicates goblet cell loss. Scale bars = 100 µm (C). Pathological scoring of samples graphed as compared with the assigned grouping of each sample: <4 (healthy control, *n* = 3); 0–4 (unaffected, *n* = 5); 4.5–7 (transitional, *n* = 6); 7.5–10 (diseased, *n* = 5).

Structural assessment of the intestinal biopsy tissue was performed to evaluate the extent of pathological tissue damage and verify the endoscopic assignment of disease severity provided by the attending surgeon. Histological assessment using hematoxylin and eosin and Alcian blue staining revealed features of CD-associated pathology compared with non-CD control tissue (Figure 1A, B). Evidence of minor inflammation and associated histoarchitectural changes were detected in the control biopsies, with evidence of low-level leukocyte infiltration with regions of inconsistent minor edema detected in several samples (66% of samples), likely due to the tissue being taken from the margins of excised tissue rather than the actual pathological section of the biopsy.

Primarily, unaffected CD samples showed evidence of slight inflammation and pathological damage, with abundant intact goblet cells consistent with the biopsy being taken from a region remote to the main lesion in the individual with CD. Biopsy specimens in the transitional CD group exhibited a range of disease severities with overall more features of severe inflammation than the corresponding unaffected CD samples; these specimens typically showed some loss of goblet cells with diminished secretion of extracellular mucus (assessed by parallel Alcian blue staining). Compared with the biopsy specimens from the unaffected and transitional CD specimens, all specimens from the diseased CD group showed evidence of severe inflammation, with features including marked goblet cell loss, consistent crypt dropout, severe edema, together with obvious leukocyte infiltration, and loss of epithelial integrity common to all specimens. The histopathological score of each sample compared with its pathological grouping (Figure 1C) illustrates the relatively high accuracy of the endoscopic evaluation prior to the actual biopsy.

### Cell Viability in the Inflamed Bowel Tissue

**Figure 2.**
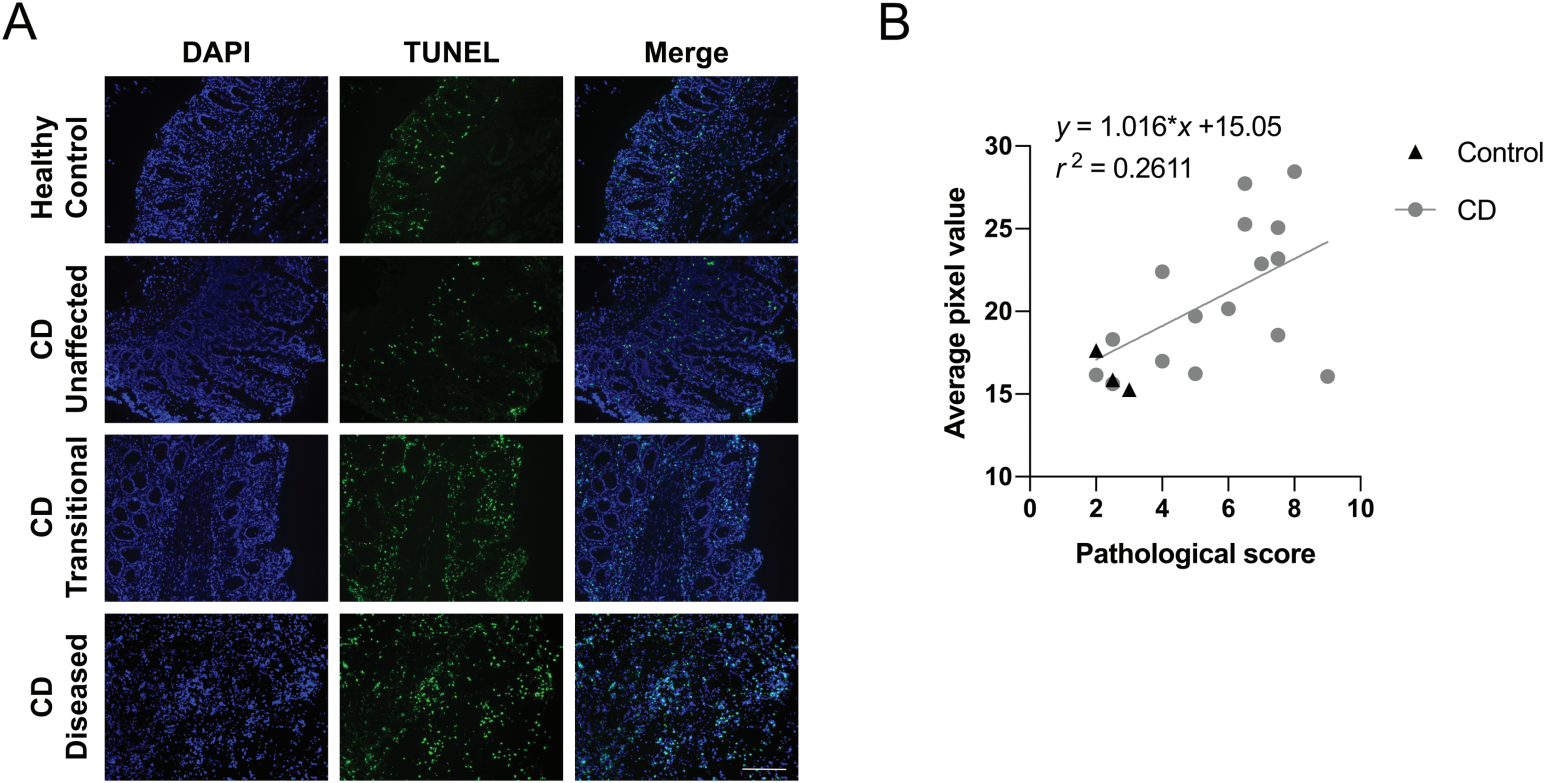
A, Representative images of TUNEL^+^ staining of frozen-sectioned healthy control and CD-affected biopsies with a fluorometric TUNEL assay. Blue: DAPI; Green: FITC—fragmented DNA. Scale bar = 100 µm. B, Linear correlation between TUNEL^+^ and average pixel value. Pearson correlation coefficient r^2^ = 0.261, *P* < .05.

Cell viability within the tissue was investigated using labeled, fragmented DNA as a marker for nonviable cells. All control specimens showed minimal loss of cell viability as judged by the low level of TUNEL^+^-staining, whereas CD specimens showed a range of cell viability that reflected the extent of inflammation in the sample, with viability decreasing with increasing disease severity (Figure 2A). Quantification of TUNEL^+^ staining revealed a significant linear correlation between pathological score and TUNEL^+^ pixel value for CD samples (*P* < .05; Figure 2B).

### Cell Infiltration in the Inflamed Bowel Tissue

Increased leukocyte infiltration is correlated with the classification and severity of disease^19^; therefore to evaluate the presence of NETs, we used antibody markers specific to components of neutrophils and NETs, including MPO, NE, and CitH3. Staining patterns for tissue imaged at 10 × 10 magnification showed varied distribution and positive immunostaining depending on disease severity of the sample being analyzed. The presence of NETs was confirmed through colocalization of DAPI and the 3 colocalizing antigens MPO, NE, and CitH3. Measurement of average pixel intensity allowed for the measurement of staining signal intensity to evaluate the density of immunostaining of each marker. Images were also taken of these areas at 40 × 10 magnification to illustrate the histological differences in areas of neutrophil infiltration where the cell structure is relatively intact, and areas where activated neutrophils had begun to undergo NET formation.

**Figure 3.**
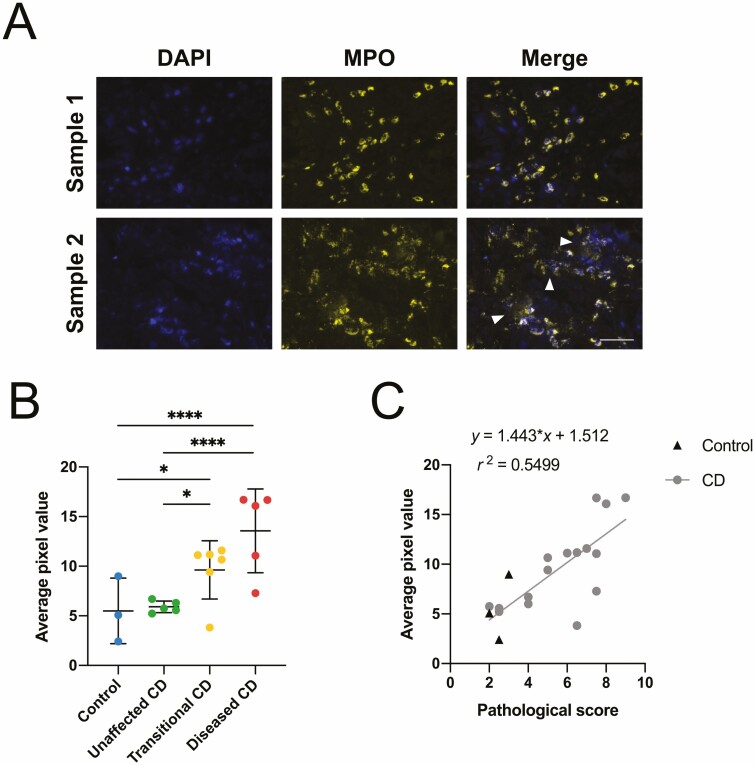
A, Representative images of neutrophil and neutrophil extracellular trap (NET) morphology observed in 7-µm sections of diseased Crohn’s patient specimens using immunofluorescence analysis of myeloperoxidase. Sample 1 represents an area of intact neutrophils in the tissue, and sample 2 represents an area where some neutrophils have undergone NET formation, indicated by white arrowheads. Scale bar = 50 µm. B, Quantification of average pixel value for *n* = 3-5 images collected per sample. Pixel value calculated and normalized for image number. Data categorized by assigned pathological grouping, presented as mean ± SD. Kruskal-Wallis test *P < .05, ****P < .0001. C, Linear correlation of immunofluorescence quantification data and pathological score. Pearson correlation coefficient r^2^ = 0.5499, *P* < .001.

The first to be investigated was MPO, an enzyme found at high concentration within neutrophils, containing much lower concentration within monocytes, and acting as a robust marker for neutrophil presence.^30^ All control samples had minor neutrophil involvement, with a few NET structures observed in the samples. Unaffected CD sections showed similar staining, with low-level neutrophil involvement and NETosis. Transitional CD samples displayed more NET production and neutrophil infiltration than the unaffected CD and control samples, and diseased CD samples displayed the most staining, with areas of intense NET release. Images generated at 40 × 10 magnification displayed characteristics of highly inflamed tissues. Distinct areas of neutrophil infiltration, with dense cytoplasmic staining of the granular enzyme were noted. Intact neutrophils retained their normal histological appearance, and areas of possible NET release with dispersed MPO^+^ immunopositivity colocalizing with diffuse areas of DAPI staining (Figure 3A). The intensity of the immune-positive staining for each sample showed significant differences to their assigned pathological (disease) severity (Figure 3B). Using the Kruskal-Wallis test for nonparametric data, significant differences were identified between control and transitional CD (*P* < .05), unaffected CD and transitional CD (*P* < .05), control and diseased CD (*P* < .0001), and unaffected CD and diseased CD (*P* < .0001). Furthermore, a significant linear correlation was determined between staining intensity and pathological score (Figure 3C), with r^2^ = 0.55 and *P* < .001.

**Figure 4.**
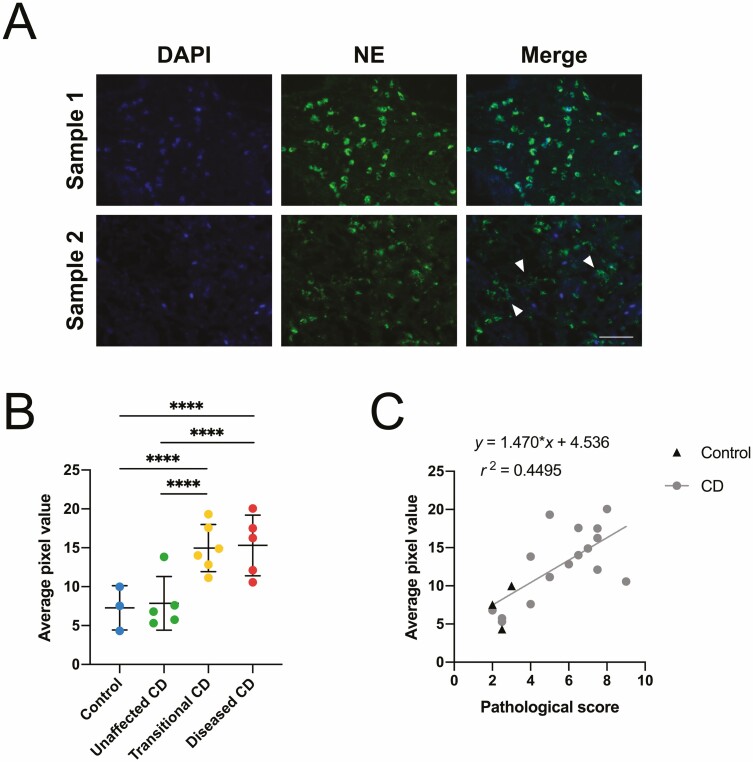
A, Representative images of neutrophil and neutrophil extracellular trap (NET) morphology observed in 7-µm sections of diseased Crohn’s patient specimens using immunofluorescence analysis of neutrophil elastase. Sample 1 represents an area of intact neutrophils in the tissue, and sample 2 represents an area where some neutrophils have undergone NET formation, indicated by white arrowheads. Scale bar = 50 µm. B, Quantification of average pixel value for *n *= 3-5 images collected per sample. Pixel value calculated and normalized for image number. Data categorized by assigned pathological grouping, presented as mean ± SD. Kruskal-Wallis test ****P < .0001. C, Linear correlation of immunofluorescence quantification data and pathological score. Pearson correlation coefficient r^2^ = 0.4495, *P* < .005.

**Figure 5.**
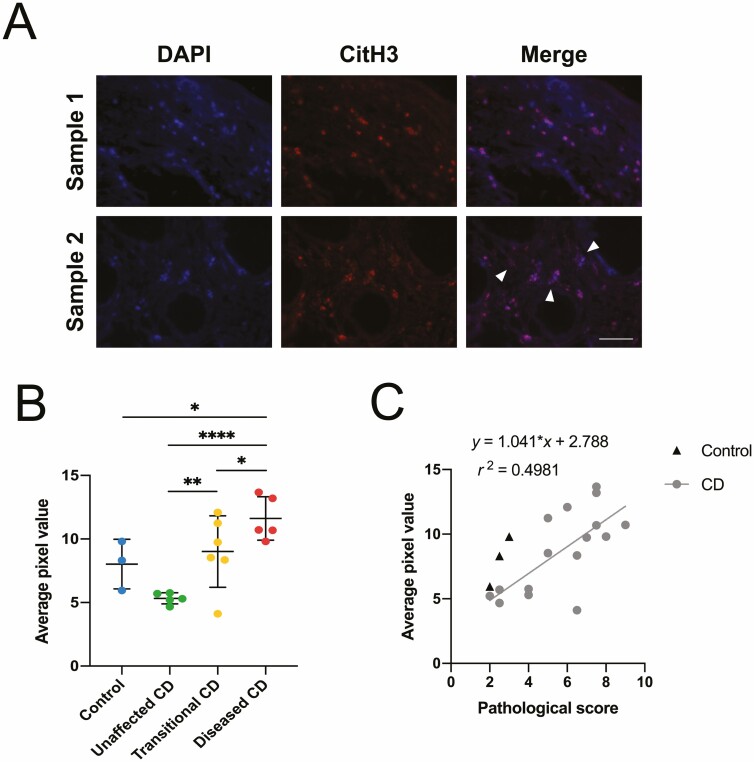
A, Representative images of neutrophil and neutrophil extracellular trap (NET) morphology observed in 7-µm sections of diseased Crohn’s patient specimens using immunofluorescence analysis of citrullinated histone H3. Sample 1 represents an area of intact neutrophils in the tissue, and sample 2 represents an area where some neutrophils have undergone NET formation, indicated by white arrowheads. Scale bar = 50 µm. B, Quantification of average pixel value for *n* = 3-5 images collected per sample. Pixel value calculated and normalized for image number. Data categorized by assigned pathological grouping, presented as mean ± SD. Kruskal-Wallis test *P < .05, **P < .01 ****P < .0001. C, Linear correlation of immunofluorescence quantification data and pathological score. Pearson correlation coefficient r^2^ = 0.4981, *P* < .005.

Neutrophil elastase is found in the same azurophilic granules as the enzyme MPO and also localizes to NET structures; thus, it can also be identified in relation to the genetic material of neutrophils via colocalization with a nuclear marker.^31^ As with the MPO-stained sections, analysis of images obtained at 10 × 10 magnification showed that NE exhibited a similar pattern of staining relating to the gradation of disease severity, with the control and unaffected CD tissue overall showing lower levels of staining than the transitional CD tissue, and diseased CD samples showing the most intense immunostaining. The images captured at 40 × 10 magnification also showed areas of dense staining around intact neutrophils and areas of possible NET release, characterized by areas of diffuse colocalization between the NE marker and DAPI (Figure 4A). Staining intensity of the marker also showed a significant correlation to assigned pathological group (Figure 4B). Again using the Kruskal-Wallis test, significant differences were found between control and transitional CD (*P* < .0001), unaffected CD and transitional CD (*P* < .0001), control and diseased CD (*P* < .0001), and unaffected CD and diseased CD (*P* < .0001). Overall, a linear correlation was established between staining data and pathological score (Figure 5C), with r^2^ = 0.4495 and *P* < .005.

The next biomarker investigated was CitH3 (Figure 5), as it represents an additional marker specific to NETosis and hence an unambiguous biomarker for neutrophils undergoing NET formation.^22^ The enzyme PAD4 converts arginine in histone H3 to citrulline, allowing decondensation of the genetic material; thus, CitH3 is specific to neutrophils that have been activated to undergo this pathway.^32^ Similar to markers MPO and NE, CitH3^+^ immunostaining intensity largely followed the gradation of disease, with control and unaffected CD samples showing low levels of staining and the diseased CD samples displaying the highest levels of staining. Imaging of sections at 40 × 10 magnification generated areas of intact neutrophils and areas of potential NET release, with areas of diffuse colocalization between the CitH3 marker and DAPI (Figure 5A). Staining intensity generated for each sample shows significant differences between their assigned pathological group (Figure 5B). Through using the Kruskal-Wallis test, this series of comparisons revealed significant differences between unaffected CD and transitional CD (*P* < .01), control and diseased CD (*P* < .05), unaffected CD and diseased CD (*P* < .0001), and transitional CD and diseased CD (*P* < .05). In addition, a linear correlation was found between staining data and numerical pathological score (Figure 5C), with r^2^ = 0.4981 and *P* < .005.

### Identifying NET Formation in the Inflamed Bowel

**Figure 6.**
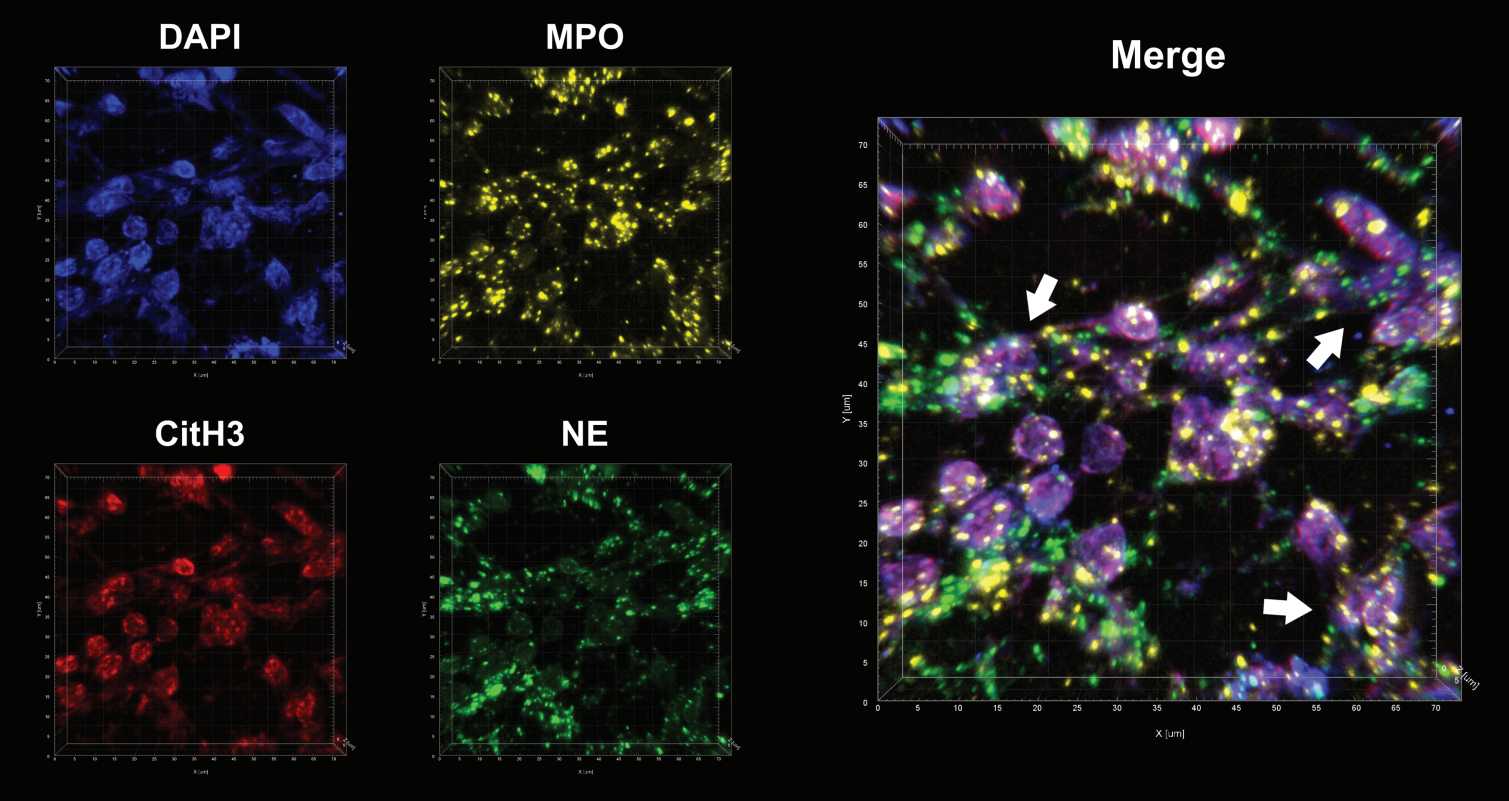
Representative immunofluorescent staining of multiplex imaging of neutrophil extracellular trap (NET) structure on diseased tissue from a Crohn’s disease patient. Tissue was labeled for myeloperoxidase (yellow), neutrophil elastase (green), and citrullinated histone (red). with nuclear marker DAPI (blue). Images taken using LSM 880 confocal microscope with Airyscan processing and then images were merged with z-stacking standard software. NET structures indicated with white arrows.

Multiplex immune-histochemical analysis was performed to analyze the distribution of multiple antibody markers on the same tissue section and was repeated for each independent specimen. Each immuno-marker was assigned a fluorophore, and the staining pattern was analyzed individually and combined to create a composite image (eg, representative image shown in Figure 6). White arrows indicate areas of NET formation, with overlapping areas of staining of each antibody marker and nuclear markers, showing a diffuse pattern. This combination of features has been observed previously in mouse gut, lung, and human brain tissue^33,34^ and is characteristic for active NETosis, with the neutrophil genetic material losing its normally dense structure and the granular enzymes translocating to the nucleus.

**Figure 7.**
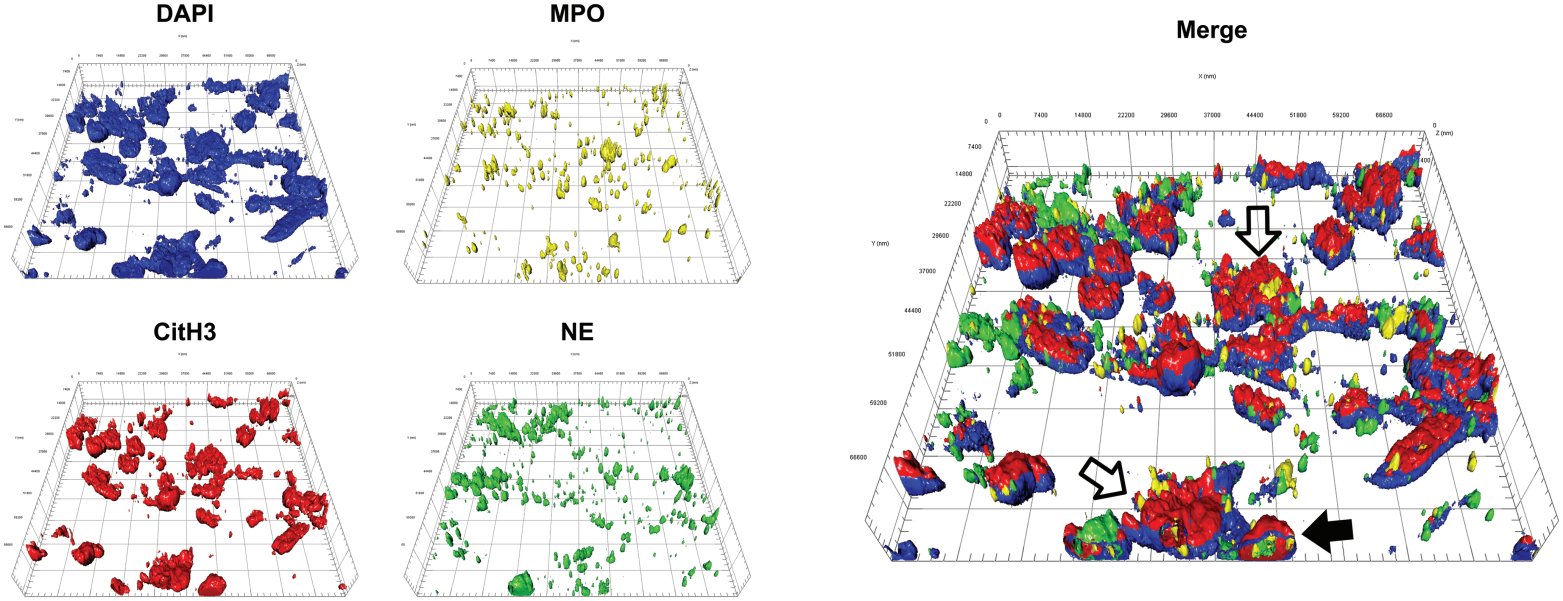
Representative 3D image of multiplex imaging of neutrophil extracellular trap (NET) structure on diseased tissue from a Crohn’s disease patient. Tissue was labeled for myeloperoxidase (yellow), neutrophil elastase (green), and citrullinated histone (red), with nuclear marker DAPI (blue). Images taken using LSM 880 confocal microscope with Airyscan processing and then merged via z-stacking using the standard software supplied with the microscope. NET structures indicated with open arrows in the merged image while neutrophil with enzymes translocated to nucleus indicated with solid (black) arrow.

In addition, image capture with the LSM880 microscope allowed for multiple image generation via the capture of successive images at sequential focal distances, thereby affording a stepwise penetration of the tissue to create a 3D (z-stacked) image, enabling real-time enhanced visualization of the NET structure in the tissue (as shown in Figure 7). Using this approach, we found that the serial images collected demonstrated colocalization of all markers in a similar pattern as previously shown in human intestinal sections.^27^ This is highlighted in the 3D rendering of the captured images, showing NET-like structures in the severely diseased tissue (Figure 7). Similar to the data shown in Figure 6, the white arrows indicate neutrophils undergoing NETosis, with evidence for decondensation of the nuclear material and translocation of the enzymes; a cross-section of a neutrophil that was undergoing this process is indicated by the black arrow. A corresponding video generated from coupling the various z-stack images highlighted the relationship between the antibody markers and the surrounding tissue architecture (as shown in Supplementary Figure 1).

## Discussion

Current research in the field of NETosis has been rapidly accelerating as researchers develop an understanding of the role that NETs play in promoting chronic inflammation. In this present study, we have demonstrated for the first time the coregistration of specific NET biomarkers—NE, MPO, CitH3, and DNA—using advanced 4-plex immunofluorescence and microscopic approaches. Data here indicated a significant positive correlation between staining intensity for all markers and increasing CD severity in samples compared with intestine histologically confirmed as healthy control. Typical NET structures were identified in each patient sample, with little evidence of NET formation determined in control samples. Although quantification of NE and MPO staining intensity was unable to distinguish between native and NETotic neutrophils, a positive correlation between CD severity and CitH3 confirmed increased NET activation in diseased samples, an outcome completely consistent with the increased neutrophil infiltration observed in these same samples. As CitH3 is the product of an irreversible process in NET formation,^22^ the increased concentration of the marker alongside other neutrophil markers indicates that there is a higher concentration of NETs and neutrophils undergoing NETosis in the samples with a greater disease severity. The positive correlation between MPO staining and extent of intestinal damage suggests a link between the extent of IBD pathology and MPO activity (and its potent enzymic product HOCl). The 3D z-stacked images also afforded NET visualization in situ, with various stages of NETosis identified in severely inflamed areas of tissue. The resultant images generated here provide a better understanding of the distribution of NETs within the cellular architecture of affected bowel tissues taken from CD patients.

The role of neutrophils in IBD pathogenesis currently remains controversial, as inhibition of neutrophil activity has been shown to have both beneficial and deleterious effects during intestinal inflammation.^5,6,35^ Recent studies with animal models of IBD using specific inhibitors of neutrophil-MPO-mediated oxidative damage have inferred protection against disease progression.^36,37^ Therefore, our data demonstrating that NETosis is evident in human CD specimens indicate neutrophil-MPO may play a role in the pathogenesis of CD.

As noted previously, NET formation has been identified in adult UC tissue but not proven in CD. Increased NET formation was shown to correlate with biopsy sampling distance from lesion tissue^38^; however, this analysis was not performed previously on CD tissue. In addition, PAD4-expressing leukocytes are elevated in UC-affected colon tissue compared with CD and control tissues. The NET markers including CitH3 were observed in UC-affected tissues; however, once again the corresponding immunohistochemistry identifying this suite of antigens in tissues was not performed on CD tissues.^39^ Proteomic analysis of NETs and examination through confocal microscopy has exhibited NET accumulation. Furthermore, lesion severity is correlated with significantly high levels of 11 neutrophil-associated proteins including calprotectin.^21^ Also, circulating NE levels are elevated in IBD patients, and this biomarker has been moderately successful at discriminating between active vs remissive cases.^40^ The NE concentration in the colonic mucosal cells of UC patients has been shown to be elevated, which was also observed to inhibit epithelial proliferation and repair of intestinal epithelial cells in vitro.^41^

Data from a mouse model of colitis demonstrated that NETs were able to cause epithelial apoptosis and disrupt the tight junctions and adherens junctions, allowing the migration of bacteria into the lamina propria.^33^ This result is consistent with other studies indicating that neutrophil depletion attenuates colitis and reduces colonic injury.^42,43^ Overall, identifying the role that NETs play in IBD pathogenesis is currently ambiguous and warrants further examination. In this human disease setting, the present correlative study helps to confirm that NETosis occurs in CD and that the extent of NET formation is related to disease severity; however, this relationship does not prove causal relationship between MPO activity and pathogenesis.

The accumulation of neutrophils and excessive NET formation at sites of inflammation is considered a primary pathogenic mechanism in several other chronic inflammatory conditions. For example, the buildup of monosodium urate crystals in gout-affected joints results in swift recruitment of neutrophils.^44^ Tophi are formed as a result of the subsequent inflammatory response, made up of macroscopically visible deposits of NETs surrounding the crystals.^45^ However, it is suggested that NETs play a role in the resolution of the disease, with NET components able to dampen the inflammatory cytokine milieu within the joint capsule, and the extracellular DNA smothering the crystals to prevent activation of inflammasomes.^46^ Increased concentration of NETs was observed in blood samples taken from type 2 diabetes patients, with prolonged hypoglycemia shown both to increase sensitivity of neutrophils to stimulation and to activate NADPH oxidase.^47^ Outcomes from studying a model of type 2 diabetes mellitus in rats has demonstrated NET formation in the retina and surrounding vasculature, suggesting a role for this structure and indeed MPO enzymic activity/MPO-mediated oxidant production in the progression of diabetic retinopathy.^47^ The inflammation associated with the autoimmune disease systemic lupus erythematosus (SLE) has also been shown to be linked to NET formation. Thus, increased levels of autoantibodies against double-stranded DNA and other nuclear components correlate with increased NETosis.^48^ The externalization of autoantigens through NET release causes further immunological activation through binding of autoantibodies to the genetic material. Completely consistent with this notion, anti-DNase antibodies and upregulation of DNase inhibitors are observed in tissue from an SLE cohort, and both were linked to Lupus nephritis.^25^

Oxidative damage associated with MPO activity has been observed to correlate with IBD severity, with both UC and CD patients exhibiting oxidation products associated with HOCl and oxidative damage mediated by H_2_O_2_.^49^ The compound AZD3241 is a specific inhibitor of extracellular MPO activity and ameliorates the effects of (dextra sodium sulfate) DSS-induced colitis in a mouse model. In this preclinical study, improved clinical scores were observed in the treatment group and in lower fecal content for markers that indicated MPO-associated damage.^36^ In addition, bacterial recombinant superoxide dismutase was shown to elevate antioxidant activity and subsequently reduce ROS damage and MPO activity in a mouse model of colitis.^50^ Depletion of plasma antioxidant concentration has been identified in IBD patients, and this appears to be most evident in patients with active disease.^51^ Interestingly, measurement of plasma thiol levels in CD patients showed that patients in a remissive state exhibited characteristically lower levels of plasma-free thiols compared with healthy controls, suggesting that patients in clinical remission still had underlying oxidative stress despite the apparent clinical resolution of inflammation.^52^

Due to the excessive production of HOCl in IBD tissue, targeting this production through the use of MPO inhibitors may be effective at ameliorating HOCl-associated inflammation. Identification of a drug that could target extracellular MPO and reduce the effect of HOCl in host-tissue damage may provide a therapeutic benefit and represent a viable alternative to current immunological-based antibody therapies that have significant unwanted side effects.^2^ Drugs that inhibit MPO have been examined in mice, with the synthetic MPO inhibitor AZD3241 shown to reduce the clinical score and pathological tissue damage in a mouse model of DSS-induced colitis.^36^ In a similar model of experimental colitis, the MPO inhibitor 4-methoxy-tempo was also shown to ameliorate histopathological damage, reduce weight loss, and reduce the density of MPO-labeled infiltrating cells.^37^ Nitroxide-containing nanoparticles designed to accumulate in the inflamed gut showed significantly lower disease activity and inflammation compared with mice given the drug through oral administration.^53^ Clinical trials in humans would be the next step in identifying whether these drugs (or analogues) are effective at inhibiting host-tissue damage associated with excessive HOCl production, without reducing the effectiveness of pathogen clearance nor causing debilitating side effects.

A major limitation in the approach to develop inhibitors of MPO and NETosis is the fact that neutrophil function is crucial for bacterial clearance, and core neutrophil bactericidal activity can facilitate pathogen invasion and colonisation, thus increasing the risk of infection and inflammation. In support of this, neutrophil depletion in mice displayed increased migration of neutrophils into the lamina propria during experimental colitis, with worsened lesion severity as a consequence.^54^ In addition, lesion severity was decreased in an NADPH oxidase knockout mouse model of experimental colitis^55^; and neutrophils isolated from the blood of CD patients were shown to have defective transepithelial migration, with significantly elevated ROS production upon stimulation.^56^ To counter this limitation, the strategic design of MPO inhibitors has focused on pharmacological approaches to inhibit extracellular MPO.^36^ There has been some success in the use of these “extracellular-specific” agents in other disease models where MPO activity is implicated in the pathogenesis.^57,58^ Whether drugs with the necessary specificity to target extracellular MPO and spare intracellular MPO phagocytic activity, can be developed at the clinical level remains to be demonstrated.

Our evaluation of NET marker quantification would have been best studied in a larger cohort of patients, and studies with equal numbers of male vs female participants are ideal. However, the specimens obtained were already present in the Biobank; these were deemed suitable for the planned work. Obtaining a complete clinical history regarding the patient demographics was also a challenge, and as such, certain variables like age, gender, and prior medication use were not available for this patient cohort.

In conclusion, we have demonstrated a positive correlation between staining intensity of NET markers and CD lesion severity in biopsy bowel tissue from adults. This is also the first time in situ quantification of NET using CitH3 as a biomarker has been performed specifically in human CD lesions. We have also used advanced imaging techniques to generate 3D images of NET formation in intestinal biopsy tissue from CD patients using specific NET markers. The data obtained indicate that there is a relationship between MPO-associated oxidative damage to host tissue and IBD lesion severity that warrants further exploration. For example, it would be useful to employ MPO knockout mice in an experimental model of IBD to ascertain whether lack of MPO activity reduces the deleterious effect of NETs in relation to disease progression.

## Supplementary Material

izab239_suppl_Supplementary_Material

izab239_suppl_Supplementary_Image

## Data Availability

The data underlying this article are available in the article and in its online supplementary material. Specific data underlying this article will be shared on reasonable request to the corresponding author.

## List of Abbreviations

CD: Crohn’s Disease
CitH3: Citrullinated Histone H3
DAPI: 4′,6-diamidino-2-phenylindole
DPX: dibutylphthalate polystyrene xylene
GI: gastrointestinal
H2O2: hydrogen peroxide
**HO** ^**•**^: hydroxyl radical
HOC: hypochlorous acid
HREC: human research ethics codes
HRP: horseradish peroxidase
IBD: inflammatory bowel disease
MBS: main beam splitter
MPO: myeloperoxidase
NADPH: nicotinamide adenine dinucleotide phosphate
NE: neutrophil elastase
NET: neutrophil extracellular trap
**O2** ^**•-**^: superoxide radical
OCT: optimal cutting temperature
PAD4: peptidyl arginine deiminase IV
PBS: phosphate buffered saline
ROS: reactive oxygen species
SLE: systemic lupus erythematosus
TBS-T: tris-buffered saline with Tween 20
TSA: tyramide signal amplification
TUNEL: Terminal Deoxynucleotidyl Transferase dUTP Nick End Labeling
UC: ulcerative colitis
