# Supplementary material for: Neutrophil Extracellular Trap Density Increases With Increasing Histopathological Severity of Crohn’s Disease
Source: Inflamm Bowel Dis. 2021 Nov 1;28(4):586–98. doi: 10.1093/ibd/izab239 (PMC9036391; doi:10.1093/ibd/izab239)
Supplement: izab239_suppl_Supplementary_Material [file izab239_suppl_supplementary_material.docx]

**Neutrophil extracellular trap density increases with increasing histopathological severity of Crohns Disease**

Angie Schroder,^1,2^ Belal Chami,^1,2^ Yuyang Liu,^1,2^ Chloe M Doyle,^1,3^ Mary El Kazzi,^1,2^ Golo Ahlenstiel,^4^ Gulfam Ahmad,^1,2^ Nimalan Pathma-Nathan,^5^ Geoff Collins,^5^ James Toh,^5^ Andrew Harman,^1,3^ Scott Byrne,^1,3^ Paul K Witting^1,2^

*^1^The University of Sydney, School of Medical Sciences, Faculty of Medicine and Health, NSW, 2006 Australia;*

*^2^ Charles Perkins Centre, The University of Sydney, NSW, 2006 Australia*

*^3^ Westmead Institute for Medical Research, Centre for Immunology and Allergy Research, Westmead, NSW, 2145 Australia*

*^4^ Western Sydney University, Westmead Clinical School and The Westmead Institute for Medical Research, Blacktown Hospital*

*^5^ Centre for Virus Research, The Westmead Institute for Medical Research, Westmead, NSW 2145 Australia and Department of Colorectal Surgery, Westmead Hospital, NSW 2145, Australia*

**Supplementary Material**

Table 1: Histopathological scoring criteria

| Score | Crypt density | Epithelial integrity | Oedema | Goblet cell loss | Leukocyte infiltration |
| --- | --- | --- | --- | --- | --- |
| 0 | Normal | Normal | None | None | None |
| 0.5 | Mostly intact  0-25% loss | Minimal shedding  0-25% loss | Minimal | Mild  0-25% loss | Mild |
| 1 | Moderate loss  26-50% loss | Moderate shedding  26-50% loss | Moderate | Moderate  26-50% loss | Moderate |
| 1.5 | Extensive loss  51-75% loss | Extensive damage  51-75% loss | Extensive | Extensive  51-75% | Extensive |
| 2 | Entire crypt dropout  76-100% loss | Complete loss **and/or** crypt rupture  76-100% loss | Transmural | Entire loss  76-100% | Transmural |


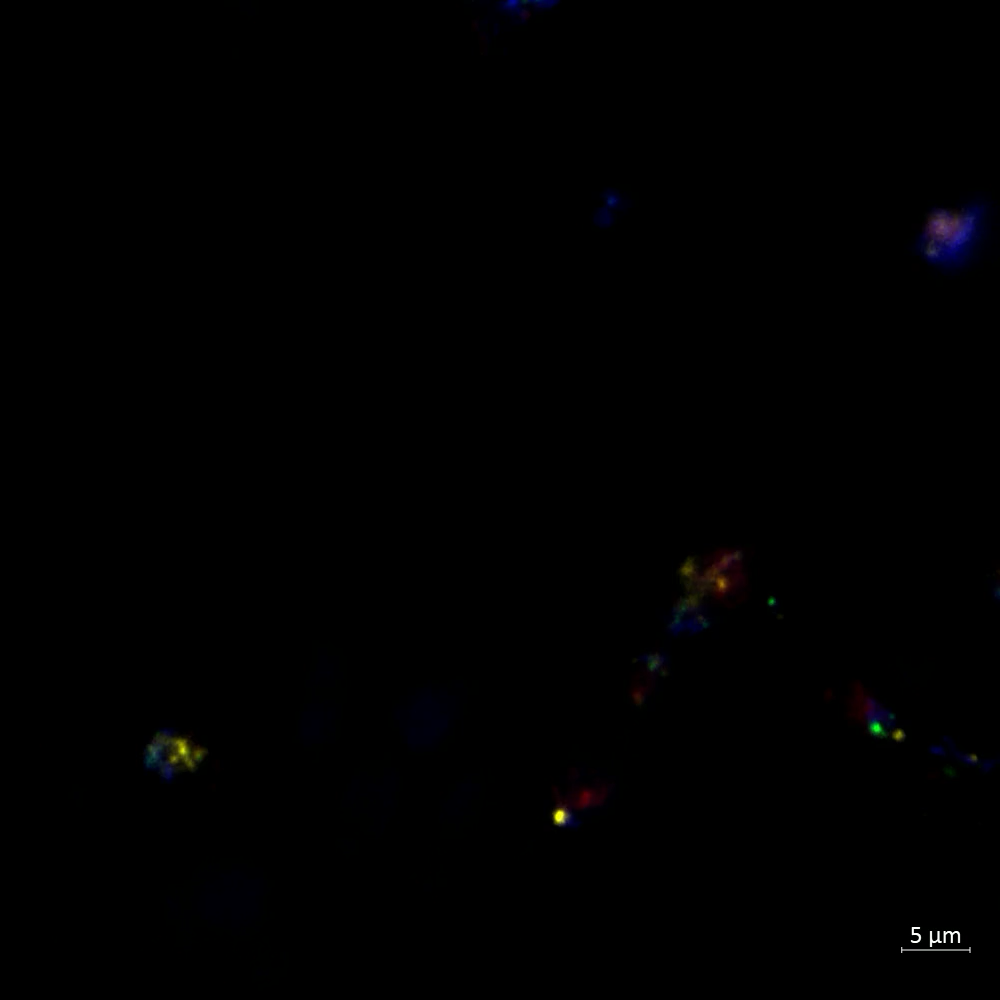


**Supplementary Figure 2.** Animated representative 3-dimensional image of multiplex imaging of neutrophil extracellular trap (NET) structure on a single section of diseased tissue from a Crohn's disease patient (to view this MP4 video please activate the link here: <https://ses.library.usyd.edu.au/handle/2123/24750>). Tissue was labelled for anti-myeloperoxidase (false coloured yellow), anti-neutrophil elastase (green) and anti-citrullinated histone (red) with nuclear staining shown with DAPI (blue). Images taken using LSM 880 confocal microscope with Airyscan processing and merged via Z-stacking. The images move upwards through the colonic tissue structure, illustrating the 3-dimenional formation of NETs within the tissue.
